# Integrated smart flock monitoring of reproductive parameters for lambing date prediction in merino sheep

**DOI:** 10.3389/fvets.2026.1937513

**Published:** 2026-09-10

**Authors:** Burak Yılmaz, Koray Tekin

**Affiliations:** Faculty of Veterinary Medicine, Department of Reproduction and Artificial Insemination, Ankara University, Ankara, Türkiye

**Keywords:** automated weighing, breeding soundness examination, estrus, mating marker, precision livestock farming, reproductive management, sheep breeding

## Abstract

**Introduction:**

Integrated flock monitoring systems may improve reproductive decision-making by linking maternal, male, and mating records within a single digital framework. This study evaluated whether a smart flock monitoring system integrating RFID identification, automated body-weight recording, mating-marker data, estrus observation, ram breeding soundness examination, semen evaluation, and pregnancy diagnosis could support lambing-date prediction under commercial Merino flock conditions.

**Methods:**

The study included 210 multiparous ewes and 11 mature rams; five rams underwent detailed breeding-soundness, semen-quality, and service-capacity evaluations. Sixty ewes underwent an 11-day progesterone-sponge synchronization protocol followed by PMSG administration and were allocated to two mating-management groups. Among routinely managed ewes, 51 formed the primary lambing-date analytical dataset, while 49 with complete natural-mating and lambing records were evaluated retrospectively using the fixed 148-day prediction rule.

**Results:**

Estrus duration showed marked individual variability. The interval from induced estrus to subsequent estrus was 16.33 ± 0.28 days in Group 1 and 19.95 ± 0.17 days in Group 2 (*p* < 0.001), although these intervals arose under different mating-management conditions and were interpreted separately. Ram service capacity was positively correlated with 45-day pregnancy rate (rs = 0.9, *p* = 0.037) and negatively correlated with sperm concentration (rs = −0.9, *p* = 0.037). Body weight and body condition score increased from pre-breeding to late gestation and declined after lambing. The mean absolute difference between estimated and observed lambing dates in the primary analytical dataset was 3.23 ± 2.47 days. In the natural-mating comparison, mean gestation duration was 147.24 ± 0.28 days, and the fixed 148-day rule produced a mean absolute prediction error of 1.37 ± 0.22 days; 77.6% of lambings occurred within ±1 day and 95.9% within ±2 days of the estimated date. Singleton pregnancy and maternal body weight were associated with absolute prediction error, whereas offspring sex was associated with gestation duration, although both regression models had limited explanatory power.

**Discussion:**

Integrated smart flock monitoring can generate biologically meaningful reproductive datasets and may support practical lambing-date prediction, but the regression findings require confirmation in larger independent populations.

## 1 Introduction

Reproductive efficiency in sheep production depends not only on successful conception and pregnancy establishment but also on effective management during late gestation and the immediate postpartum period. Lamb mortality within the first 48 h after birth remains a major source of productivity loss worldwide (1). Early lamb deaths are commonly associated with dystocia, perinatal stress, hypothermia, starvation, delayed colostrum intake, and mismothering rather than infectious causes alone (2). Because many of these risk factors are management related, timely supervision around parturition is essential for improving neonatal survival and flock productivity. Epidemiological evidence indicates that appropriate nutritional management of late-gestation ewes, structured health programs, veterinary involvement, and systematic record keeping are strongly associated with improved lamb survival under commercial conditions (3–5).

Accurate prediction of lambing date is essential for optimizing periparturient supervision and intervention. Targeted monitoring during lambing enables timely assistance in cases of dystocia, prevention of hypothermia, and timely colostrum intake, each of which is a critical determinant of neonatal viability (1). However, under commercial conditions, biological variability in estrus duration, mating distribution, litter size, and maternal body reserves reduces the precision of lambing date estimation when based solely on mating records or average gestation length (6, 7). Even small deviations between estimated and actual lambing dates may compromise the efficiency of targeted supervision, particularly in large flocks. Sensor-based systems that monitor ewe-ram interactions have demonstrated potential for improving lambing date prediction (8, 9). Nevertheless, these approaches primarily rely on mating activity and rarely incorporate maternal physiological indicators or detailed measures of male reproductive performance, thereby limiting their ability to account for biological variability in gestational dynamics.

Estrus characteristics may be influenced by mating frequency and ram exposure (10), while ejaculation frequency and service capacity can affect semen quality and fertilization outcomes (11, 12). Consequently, breeding soundness evaluation and appropriate pre-breeding ram management are critical components of flock reproductive performance (13–16). Male reproductive parameters have rarely been incorporated into predictive models of lambing distribution under commercial flock conditions.

Maternal body weight and body condition score (BCS) are established indicators of energy balance and reproductive resilience, influencing estrus expression and pregnancy outcomes (17, 18). Continuous monitoring of these parameters may therefore enhance predictive models of gestational timing. However, most sensor-based lambing prediction studies focus predominantly on behavioral proximity and do not systematically incorporate maternal physiological status.

Precision livestock farming technologies now enable continuous, individual-based reproductive monitoring through automated identification systems, RFID tracking, longitudinal body weight recording, and centralized digital data integration (19–21). Automated estrus detection systems have demonstrated reliability as alternatives to visual observation (22, 23). Together, these technological advances create opportunities to integrate mating activity, maternal condition, and ram performance within unified predictive frameworks. However, evidence supporting the practical implementation of fully integrated precision reproductive monitoring systems for lambing date prediction in commercial sheep enterprises remains limited, particularly in regions where technological adoption is still developing (24).

To date, limited evidence is available on the practical value of combining maternal physiological indicators, ram service capacity, spermatological traits, and verified mating activity within a unified digital framework for lambing-date prediction under commercial flock conditions.

The objective of this study was to evaluate whether integrated precision monitoring of maternal, male, and reproductive parameters under commercial Merino flock conditions could support lambing-date estimation and provide an individual-animal framework for reproductive monitoring. We further examined whether maternal and offspring characteristics were associated with absolute prediction error and gestation duration.

## 2 Materials and methods

### 2.1 Animals and flock management

The study was initiated in July 2022 with RFID ear tagging, adaptation of the animals to the smart sheep-handling system, entry of individual animal data into the digital management application, and baseline health and reproductive examinations, including breeding soundness examinations of the rams. The synchronization component was subsequently initiated during the natural breeding season (October–November 2022). The study involved 210 multiparous Merino ewes from a commercial flock located in Gölbaşı, Ankara, Türkiye (Flock No: TR060000669309). The commercial flock maintained 11 mature rams, of which five (>2 years of age) were selected for the detailed breeding-soundness, semen-quality, and service-capacity evaluations performed in the present study. The overall ram-to-ewe ratio was therefore approximately 1:19.

Of the 210 ewes included in the study, 60 were enrolled in the synchronization component and were initially allocated equally to two mating-management groups (*n* = 30 per group). During subsequent review of the camera recordings, two ewes originally allocated to Group 2 were found to have been inadvertently mated by a ram assigned to Group 1 during the induced estrus period. Because Group 2 was designed to remain unmated during the induced estrus, these two ewes were reclassified according to the mating exposure actually received, resulting in post-exposure group sizes of 32 ewes in Group 1 and 28 ewes in Group 2. Four additional ewes in Group 2 were excluded from the subsequent-estrus evaluation because of vaginitis, absence of clear estrus behavior, or failure to mate, leaving 24 ewes available for the Group 2 follow-up evaluation. Sample sizes for individual outcome analyses are reported in the corresponding tables.

The remaining 150 ewes were managed under routine reproductive-management conditions. Records required for the primary descriptive evaluation of gestation duration and lambing-date prediction error were available for 51 ewes. Of these, 47 had complete data for all variables included in the multiple regression models and were therefore included in the primary regression analyses. In addition, natural-mating records from 53 routinely managed ewes were screened for a retrospective comparison analysis. Forty-nine ewes had complete records for both mating date and subsequent lambing date and were included in this comparison, whereas four were excluded because one or both dates were unavailable. Eligibility for the comparison analysis was based solely on availability of mating and lambing records, and animals were not selected according to gestation duration or prediction outcome. These animals were outside the synchronization cohort. Recorded mating dates in the natural-mating comparison ranged from 16 June to 15 October 2022. This comparison was a retrospective analytical subset of the routine-management population and was not treated as an independent validation population. Animal allocation, exclusions, and analytical subsets are summarized in Supplementary Figure S3.

Individual reproductive and productivity records were maintained throughout the study. Ewes received a daily ration consisting of 700 g barley, 250 g corn meal, vitamin–mineral supplements, salt licks, and high-quality hay provided ad libitum. Rams were supplemented with 1,250 g barley and hay prior to the breeding season. Lambs were fed commercial growth feed (18.5% crude protein), barley straw, and milk replacer (16% crude protein) according to standard flock-management practices.

### 2.2 Animal identification

All animals were individually identified using radio-frequency identification (RFID) ear tags (Allflex®, France) prior to the onset of reproductive monitoring to ensure consistent longitudinal data collection. Ear tagging was performed under hygienic conditions. The external ear surface was cleaned and disinfected before tag application to minimize local irritation or infection risk. Tags were placed vertically in the middle third of the ear pinna, avoiding major blood vessels and cartilage ridges, in accordance with manufacturer guidelines and animal welfare standards. Following placement, tag retention and signal readability were verified. RFID identification enabled automated recording and linkage of individual animal data within the iLivestock digital management platform (Scotland, UK), including animal ID, age, sex, body weight, body condition score (BCS), mating records, reproductive examinations, estrus history, and pregnancy outcomes.

### 2.3 Smart flock monitoring system

A smart flock handling and monitoring system was installed at the pasture exit gate to allow voluntary passage of animals through a single-animal channel. This configuration enabled standardized data acquisition while minimizing handling stress and disruption to flock behavior. Animals were gradually acclimatized to the system using positive reinforcement (placement of straw, dried grass, and mineral blocks near entry and exit points), which reduced avoidance behavior and promoted consistent voluntary use. The handling unit (Alligator system) consisted of a double-sided structural frame with a controlled entrance gate, exit gate, and internal selection door (Supplementary Figure S1). This design allowed controlled animal flow, temporary individual confinement for measurements, and optional sorting for reproductive examinations or management procedures.

RFID ear tags were automatically detected upon entry using an AWR 300 tag reader. Live body weight was recorded via an integrated electronic weighing platform, and data were automatically transferred through an Eweigh Bluetooth converter for real-time registration. Weight records were directly linked to RFID identifiers within the iLivestock software, enabling longitudinal tracking of body weight across reproductive stages. High-definition cameras positioned within the unit captured animal movement and mating-related behaviors. Rams were equipped with mating markers, and mating events were confirmed through combined visual recordings and marking evidence, allowing objective assessment of service capacity and temporal mating distribution. All data were integrated into a centralized digital platform (iLivestock software). Data acquisition occurred through automated logging and manual entry via smartphone interfaces when required. The system enabled real-time visualization and retrospective analysis of reproductive and physiological parameters. The integrated framework synchronized reproductive, physiological, and behavioral datasets at the individual level for subsequent statistical modeling.

In addition to reproductive monitoring, the practical handling performance of the system was recorded descriptively by comparing routine management sessions performed with and without the smart handling unit. Time requirement, number of workers, and number of sheep handled per session were summarized in Supplementary Figure S2.

### 2.4 Breeding soundness examination of rams

Breeding soundness examinations were performed 2 months before the breeding season and repeated at the onset of breeding activity. Body condition score was assessed using a 5-point scale based on palpation of the lumbar region (17). A complete physical examination of the reproductive tract was performed, including evaluation of the penis, prepuce, scrotum, testes, and epididymides. Scrotal circumference and testicular dimensions were measured and recorded (13).

At each breeding soundness examination, a single ejaculate was collected from each ram using an artificial vagina in the presence of an estrous ewe. Ejaculate volume was measured directly in a graduated conical tube. Progressive sperm motility was evaluated by phase-contrast microscopy using a heated stage maintained at 37 °C and expressed as the percentage of progressively motile spermatozoa. Sperm concentration was determined using an AccuCell photometer calibrated for ovine and caprine semen (IMV Technologies, L’Aigle, France). Sperm morphology was assessed following SpermBlue staining, with 60 spermatozoa evaluated per ejaculate and classified as morphologically normal or abnormal (25, 26).

### 2.5 Synchronization, estrus detection, and mating

During the natural breeding season, 60 ewes underwent an 11-day synchronization protocol and were initially allocated equally to two mating-management groups (*n* = 30 per group). As described in Section 2.1, two ewes initially allocated to Group 2 were subsequently reclassified to Group 1 after camera recordings confirmed mating during the induced estrus period, resulting in post-exposure group sizes of 32 ewes in Group 1 and 28 ewes in Group 2.

Both groups received the same FGA-eCG synchronization protocol so that the hormonal stimulus was held constant; the intended experimental contrast was mating exposure during the induced estrus. Group 2 was therefore synchronized but intentionally left unmated during the induced estrus to permit observation of the subsequent spontaneous cycle. No prospectively untreated third group was included in the synchronization component.

Group 1: Ewes were subjected to controlled natural mating at 36, 48, and 72 h after sponge removal. Rams were equipped with mating markers, and mating events were monitored using the integrated camera system. A mating event was considered confirmed based on combined video observation and the presence of a corresponding mating-marker mark on the ewe. In ewes that did not establish pregnancy, the interval from the induced estrus to the subsequent estrus was recorded.

Group 2: Mating was intentionally omitted during the induced estrus. Free natural mating commenced on day 15 after sponge removal and continued until day 21. Rams were equipped with mating markers, and mating events were monitored using the integrated camera system and mating-marker evidence. The interval from the induced estrus to the subsequent spontaneous estrus was recorded. Four Group 2 ewes were excluded from the subsequent-estrus evaluation because of vaginitis, absence of clear estrus behavior, or failure to mate, leaving 24 ewes available for the follow-up evaluation.

Thus, the recorded interval represented return to estrus after controlled mating in nonpregnant ewes in Group 1, whereas it represented the interval from an unmated induced estrus to the subsequent spontaneous estrus in Group 2. Because these intervals arose under different mating-management conditions, they were interpreted separately.

The synchronization protocol consisted of intravaginal sponges containing 20 mg flugestone acetate (Chronogest®, MSD, USA) for 11 days. At sponge removal, all ewes received 500 IU equine chorionic gonadotropin, formerly termed pregnant mare serum gonadotropin (eCG/PMSG; Hipra, Türkiye). A single dose was administered because all ewes weighed more than 45 kg. Estrus detection was based on behavioral signs, including tail wagging, standing to be mounted, courtship behavior, vaginal discharge, and vulvar edema (27).

### 2.6 Reproductive parameters

Pregnancy diagnosis was performed at 21 and 45 days after mating by transabdominal ultrasonography using an Esaote Tringa Linear ultrasound scanner (Esaote, Italy) (28).

The following reproductive parameters were evaluated: estrus response, pregnancy rate, gestation length, lambing rate, litter size, pre-weaning mortality, and service capacity (29, 30).

Estrus response was defined as the proportion of synchronized ewes exhibiting behavioral estrus following sponge removal. Pregnancy rate was calculated as the percentage of ewes diagnosed pregnant by ultrasonography at 21 and 45 days after mating. Lambing rate was defined as the proportion of ewes that lambed relative to the number of ewes exposed to rams. Litter size was expressed as the number of lambs born per ewe lambing. Pre-weaning mortality was calculated as the proportion of liveborn lambs that did not survive until weaning.

A service-capacity index was calculated for each ram as the number of confirmed mating events divided by the number of estrus-active ewes assigned to that ram during the observation period. Each ram had access only to a predefined group of selected ewes, allowing confirmed mating events and subsequent pregnancy outcomes to be attributed to the corresponding ram. Mating events were confirmed using mating-marker evidence and video recordings obtained from the integrated smart flock monitoring system. The index was used as an indicator of ram sexual activity and mating performance under field conditions, consistent with established principles of ram breeding-soundness and performance evaluation (13, 14).

For lambing-date estimation, the date of the first confirmed mating was used as the reference mating date. A mating was considered confirmed when it was documented by the integrated camera system and supported by a corresponding mating-marker mark on the ewe. The estimated lambing date was calculated by adding a fixed interval of 148 days to the first confirmed mating date. This interval was selected as a practical prediction reference based on published ovine gestation lengths close to 148 days (31); in naturally cycling Merino ewes, a 148-day interval also predicted 90.2% of lambing dates within ±6 days (9). Signed prediction error was calculated as the estimated lambing date minus the observed lambing date; therefore, negative values indicated that lambing occurred later than estimated, whereas positive values indicated that lambing occurred earlier than estimated. Absolute prediction error was calculated as the absolute value of the signed prediction error.

For the natural-mating comparison analysis, the mating date recorded in the routine reproductive records was used as the reference date, and the estimated lambing date was calculated by adding 148 days to this date. The same fixed 148-day prediction rule was applied without modification. Prediction performance was assessed using mean signed prediction error, mean absolute prediction error (MAE), root mean square error (RMSE), and the proportions of ewes lambing on the exact predicted date and within ±1 and ±2 days of the predicted date. This analysis was intended as a within-flock comparison of the fixed interval and did not constitute independent external validation.

### 2.7 Statistical analysis

Data distribution and homogeneity of variance were assessed using the Shapiro–Wilk and Levene tests, respectively. An *a priori* power analysis indicated that a minimum sample size of 60 ewes was required for the synchronization component; accordingly, 60 ewes were initially enrolled. Descriptive statistics are presented as mean ± standard error (SE). Independent-samples t-tests were used to compare continuous variables between independent groups. Categorical reproductive outcomes were compared using the chi-square test or Fisher’s exact test, as appropriate. Spearman’s rank correlation analysis was used to evaluate associations between ram service capacity and reproductive or spermatological parameters.

Multiple linear regression analyses were performed to identify predictors of absolute lambing-date prediction error and gestation duration. For lambing-date prediction analyses, reproductive records from the routinely managed population were screened for data availability. Records required for the descriptive evaluation of gestation duration and lambing-date prediction error were available for 51 ewes. Of these, 47 had complete data for all variables included in the multiple regression models and were therefore included in the regression analyses.

For the regression analyses, an additional retrospective sample-size assessment was performed using four predictors, an α level of 0.05, and a target statistical power of 80%. Based on the observed effect sizes of the absolute prediction-error model (*R*^2^ = 0.205; Cohen’s *f*^2^ = 0.258) and the gestation-duration model (*R*^2^ = 0.151; Cohen’s *f*^2^ = 0.178), the estimated sample sizes required to achieve 80% power were approximately 52 and 73 ewes, respectively. Because complete predictor data were available for 47 ewes, the regression analyses were considered exploratory, particularly for the gestation-duration model.

For the retrospective natural-mating comparison, 49 ewes with complete mating and lambing records were included. Prediction performance was summarized using signed prediction error, MAE, RMSE, and the proportions of observations falling on the exact predicted date and within ±1 and ±2 days of the predicted date. No separate regression model was fitted for this comparison. As a sensitivity analysis, prediction dates were additionally recalculated using fixed mating-to-lambing intervals ranging from 146 to 152 days. For each interval, the proportions of lambings occurring within ±1, ±2, and ±3 days of the predicted date were calculated. The predefined 148-day interval remained the primary reference and was not selected or optimized on the basis of this sensitivity analysis.

Predictor variables were selected *a priori* based on biological relevance. Litter size and offspring sex were entered as categorical variables, with twin pregnancy and female offspring as the reference categories. Because absolute prediction error is bounded at zero and may not inherently follow a normal distribution, the suitability of linear regression for this outcome was specifically evaluated through residual diagnostics. Model assumptions were evaluated using plots of standardized residuals against standardized predicted values, normal Q–Q plots, the Durbin–Watson statistic, variance inflation factors, standardized residuals, leverage values, and Cook’s distance. Longitudinal body weight and BCS data were summarized descriptively. All analyses were conducted using SPSS version 23.0 (IBM Corp., USA). Statistical significance was set at *p* < 0.05 with a 95% confidence interval.

### 2.8 Ethical approval

The study was approved by the Ankara University Local Ethics Committee for Animal Experiments on 10 November 2020 (approval no. 53184147–50.04.04/316312). Written permission was also obtained from the commercial farm. All procedures were conducted in accordance with relevant national and institutional animal-welfare regulations.

## 3 Results

### 3.1 Handling efficiency of the smart flock monitoring system

Descriptively, sessions performed using the smart handling unit required less time and fewer workers and involved more sheep per session than conventional handling sessions. Compared with conventional handling, use of the system reduced the average handling time from 4.89 ± 2.60 h to 2.24 ± 1.23 h and reduced labor requirement from 5.15 ± 1.68 to 3.43 ± 0.84 workers. The average number of sheep handled per session increased from 77.33 ± 60.72 to 118.46 ± 70.27. These descriptive handling-efficiency data are presented in Supplementary Figure S2.

### 3.2 Body condition score and body weight dynamics

**Figure 1:**
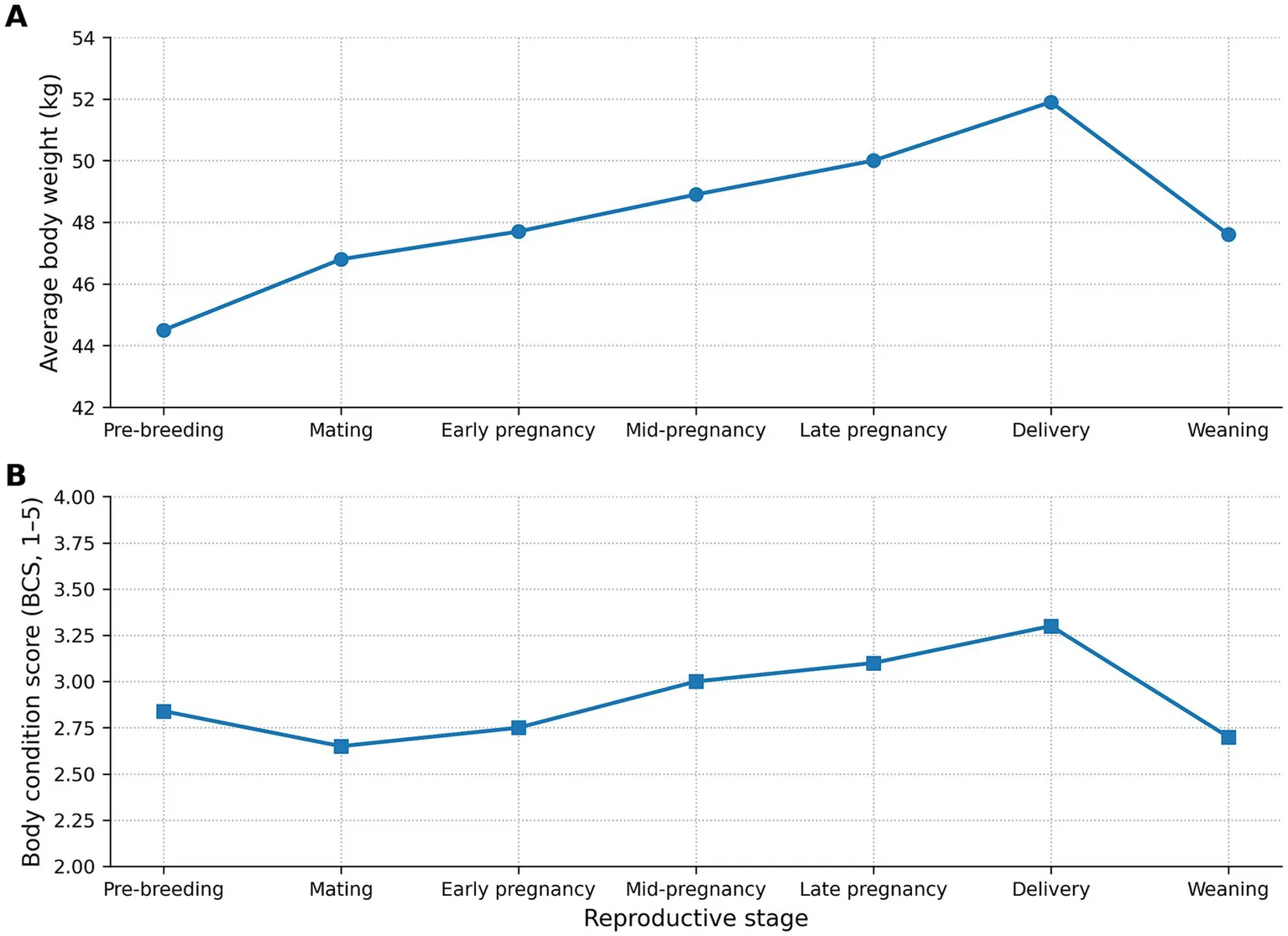
Mean body weight and body condition score across reproductive stages in Merino ewes. **(A)** Mean body weight (kg). **(B)** Mean body condition score (BCS; 1–5 scale). Values are shown for the pre-breeding, mating, early-pregnancy, mid-pregnancy, late-pregnancy, delivery, and weaning stages.

BCS did not differ significantly between groups at day 21 or day 45 (*p* > 0.468), with mean values ranging between 2.5 and 2.75. Across litter sizes, median BCS remained approximately 2.5, with a slight but non-significant increase to 3.0 in ewes carrying single lambs (*p* = 0.792). Across reproductive stages, both body weight and BCS increased from pre-breeding (BCS: 2.84 ± 0.47; BW: 44.5 ± 8.55 kg) to delivery (BCS: 3.3 ± 0.47; BW: 51.9 ± 8.59 kg), as illustrated in Figure 1. Following parturition, both parameters declined at weaning (BCS: 2.7 ± 0.48; BW: 47.6 ± 8.14 kg; Figure 1).

### 3.3 Synchronization and estrus behavior

**Figure 2:**
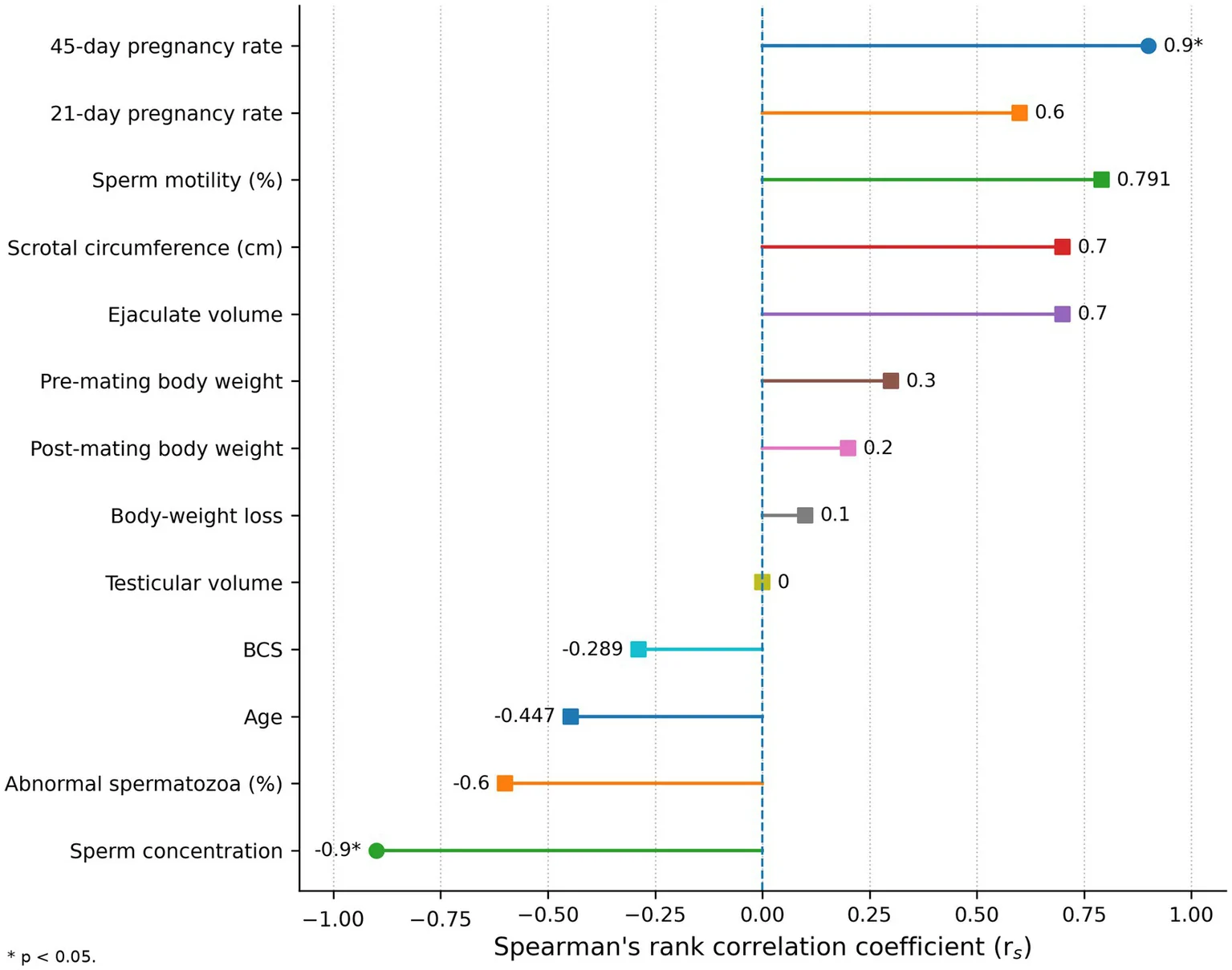
Frequency of observed estrus behaviors in synchronized ewes. Bars represent the percentage of pregnant ewes exhibiting urination, vulvar hyperemia, standing heat, courtship behavior, mild vulvar edema, marked vulvar edema, and tail wagging during estrus monitoring.

Following reclassification according to actual mating exposure, Group 1 comprised 32 ewes and Group 2 comprised 28 ewes. Four Group 2 ewes were subsequently excluded from the subsequent-estrus follow-up, leaving 24 evaluable ewes. Observed estrus behaviors included tail wagging, courtship behavior, standing heat, urination, vulvar hyperemia, and vulvar edema. No significant association was detected between specific estrus behaviors and pregnancy outcome. Among pregnant ewes, behavioral frequencies were distributed as follows: urination (57.4%), vulvar hyperemia (51.85%), standing heat (44.44%), courtship behavior (42.59%), mild vulvar edema (25.92%), marked vulvar edema (22.22%), and tail wagging (29.62%) (Figure 2). Mean estrus duration was 33.71 ± 2.62 h (range: 13–72 h) for Group 1 during the induced estrus and 37.64 ± 2.07 h (range: 14–52 h) for Group 2 during the subsequent spontaneous estrus. The numerical difference was not statistically significant (*p* = 0.277; Table 1). Because these observations were obtained under different cycle and mating-management conditions, the comparison was interpreted descriptively rather than as a direct treatment effect.

**Table 1:** Estrus duration and interval from induced estrus to subsequent estrus in synchronized ewes.

Variable	Group	*n*	Mean ± SE	Range	*p*-value
Estrus duration (h)	Group 1	32	33.71 ± 2.62	13–72	0.277
	Group 2	24	37.64 ± 2.07	14–52	
Interval from induced estrus to subsequent estrus (days)	Group 1	12	16.33 ± 0.28	15–18	<0.001
	Group 2	24	19.95 ± 0.17	18–21	

Values are presented as mean ± SE. Group 1: 11-day synchronization followed by natural mating; Group 2: 11-day synchronization, omission of the first estrus, followed by natural-cycle mating.

The interval from induced estrus to subsequent estrus was 16.33 ± 0.28 days in Group 1 and 19.95 ± 0.17 days in Group 2; however, the intervals represented different mating-management conditions and were therefore interpreted separately (Table 1).

### 3.4 Service capacity and spermatological parameters

**Figure 3:**
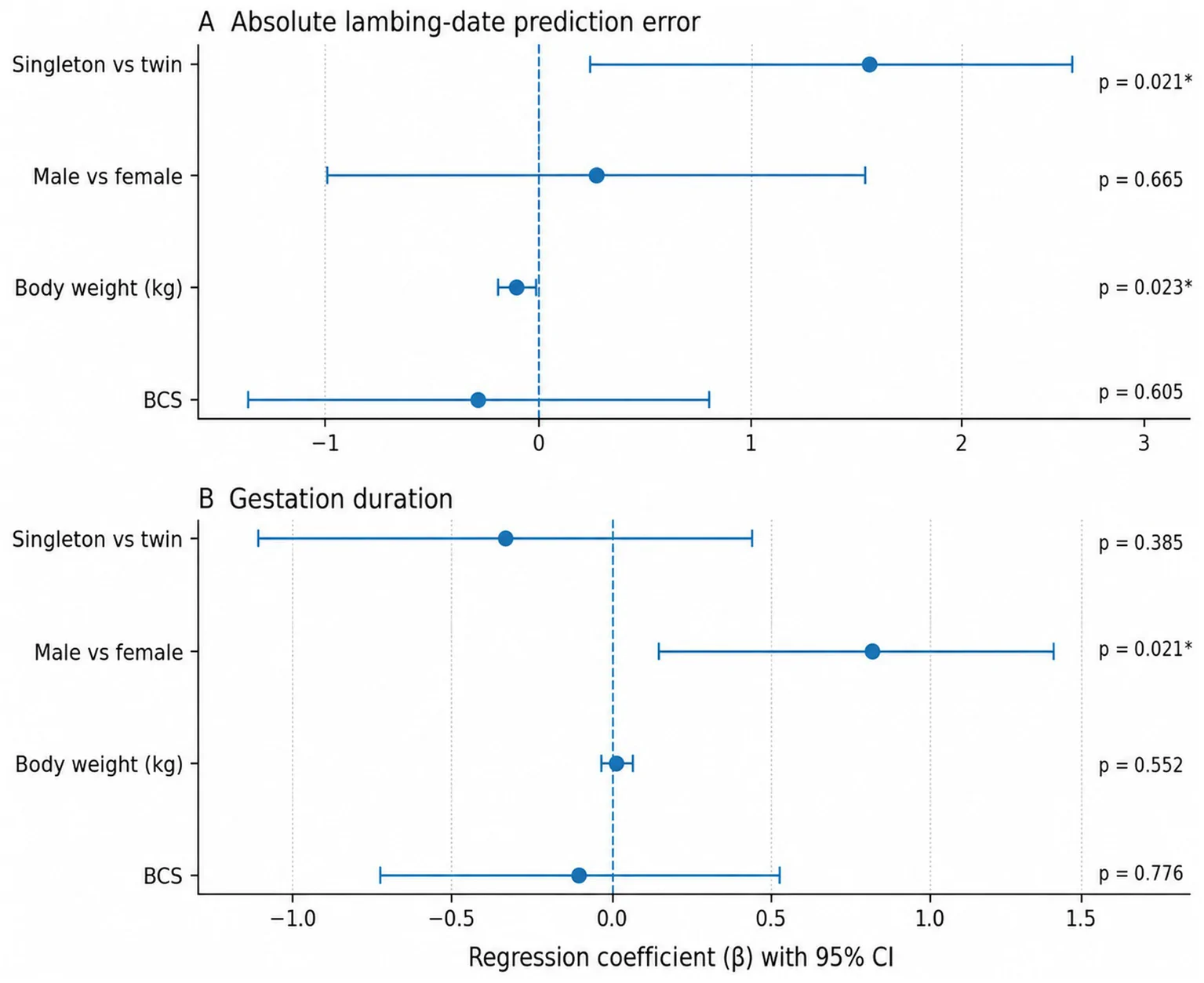
Correlations between ram service capacity and reproductive or spermatological parameters. Spearman’s rank correlation coefficients show the associations between the service-capacity index and 45-day pregnancy rate, sperm concentration, scrotal circumference, ejaculate volume, progressive sperm motility, abnormal spermatozoa, age, and body condition score in five rams. Positive coefficients indicate direct associations, whereas negative coefficients indicate inverse associations. Asterisks indicate statistically significant correlations (*p* < 0.05).

Service capacity demonstrated a strong positive correlation with 45-day pregnancy rate (rs = 0.9, *p* = 0.037) and a strong negative correlation with sperm concentration (rs = −0.9, *p* = 0.037), as illustrated in Figure 3.

Scrotal circumference and ejaculate volume showed positive but non-significant correlations with service capacity (rs = 0.7). Progressive sperm motility also showed a positive, non-significant association (rs = 0.791). The proportion of abnormal spermatozoa demonstrated a moderate negative, non-significant correlation with service capacity (rs = −0.6). Age and BCS showed no significant associations (Figure 3).

### 3.5 Early-pregnancy body weight and litter size

No significant differences were observed in ewe body weight at day 21 of pregnancy between non-pregnant ewes (43.81 ± 1.45 kg) and pregnant ewes (46.32 ± 1.58 kg; *p* = 0.256). Similarly, no significant difference was detected at day 45 (non-pregnant: 45.03 ± 1.53 kg; pregnant: 44.61 ± 1.54 kg; *p* = 0.846). Body weight did not differ significantly according to litter size (*p* = 0.194), although ewes carrying twins showed numerically higher body weights (48.5 ± 1.54 kg) compared to those carrying singletons or non-pregnant ewes.

### 3.6 Lambing-date prediction

The descriptive lambing-date dataset included 51 ewes with available gestation and lambing records. Of these, 47 had complete data for all predictor variables and were included in the multiple regression analyses.

**Figure 4:**
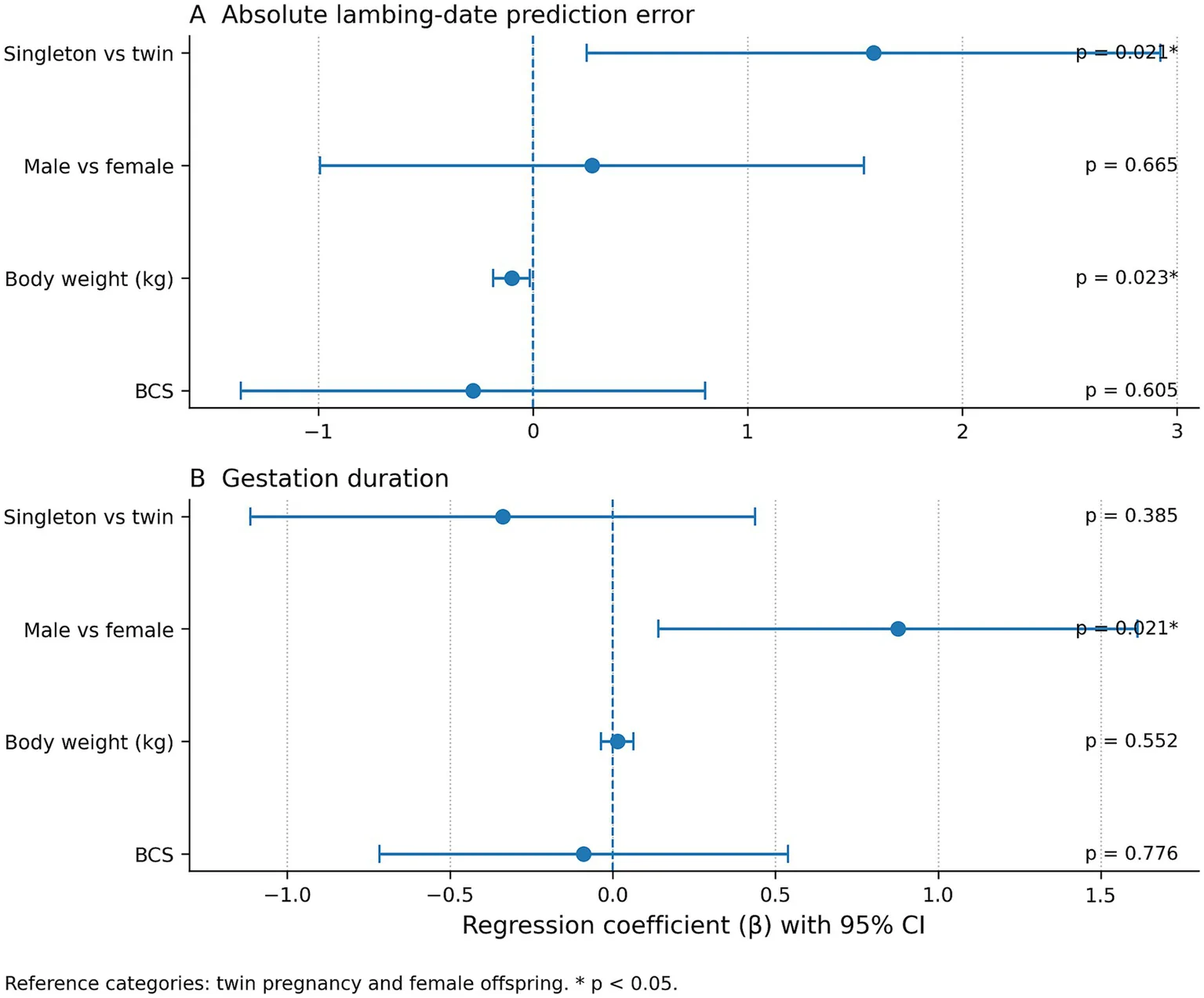
Multiple linear regression coefficients for predictors of lambing-date prediction error and gestation duration. **(A)** Absolute lambing-date prediction error. **(B)** Gestation duration. Points represent regression coefficients, and horizontal bars represent 95% confidence intervals. The vertical dashed line represents no effect. Twin pregnancy and female offspring were used as the reference categories. Asterisks indicate statistically significant effects (*p* < 0.05).

Multiple regression analysis identified significant predictors of absolute lambing-date prediction error and gestation duration. In the prediction-error model, the intercept was 7.980 days. Singleton pregnancies were associated with a greater absolute prediction error than twin pregnancies (B = 1.587, *p* = 0.021), whereas body weight was inversely associated with prediction error (B = −0.100, *p* = 0.023). The overall mean absolute prediction error was 3.23 ± 2.47 days. Regression coefficients and confidence intervals are presented in Figure 4 and Table 2.

**Table 2:** Multiple linear regression models for absolute lambing-date prediction error and gestation duration.

Outcome	Predictor	*B*	*SE*	*t*	95% CI	*p*-value
Absolute lambing-date prediction error	Intercept	7.980	2.471	3.230	2.994 to 12.967	0.002
	Singleton pregnancy	1.587	0.662	2.398	0.251 to 2.923	0.021
	Male offspring	0.274	0.628	0.437	−0.993 to 1.542	0.665
	Body weight (kg)	−0.100	0.042	−2.363	−0.185 to −0.015	0.023
	BCS	−0.279	0.535	−0.522	−1.360 to 0.801	0.605
Gestation duration	Intercept	146.973	1.434	102.457	144.078 to 149.868	<0.001
	Singleton pregnancy	−0.337	0.384	−0.878	−1.113 to 0.438	0.385
	Male offspring	0.876	0.365	2.403	0.140 to 1.612	0.021
	Body weight (kg)	0.015	0.024	0.600	−0.035 to 0.064	0.552
	BCS	−0.089	0.311	−0.287	−0.717 to 0.538	0.776

Reference categories were twin pregnancy and female offspring. Model *R*^2^ was 0.205 for absolute lambing-date prediction error and 0.151 for gestation duration. B, regression coefficient; SE, standard error; CI, confidence interval; BCS: body condition score.

Body weight had a significant inverse association with absolute lambing-date prediction error, with each 1 kg increase associated with a 0.1-day reduction in prediction error (*p* = 0.023). Offspring sex did not significantly affect prediction error (male offspring: B = 0.274, *p* = 0.665), and BCS was not a significant predictor (*p* = 0.605). In the gestation-duration model, the intercept was 146.973 days. Male offspring were associated with 0.876-day longer gestation compared with female offspring (*p* = 0.021). Model *R*^2^ values were 0.205 for absolute prediction error and 0.151 for gestation duration. Residual diagnostics did not indicate major violations of linear-regression assumptions. Standardized residuals showed no clear pattern against predicted values, and normal Q–Q plots indicated approximate normality. Durbin–Watson statistics were close to 2, suggesting no relevant residual autocorrelation. Variance inflation factors were below the accepted threshold, and no observations showed excessive leverage or Cook’s distance. Diagnostic plots are presented in Supplementary Figure S4. As these assessments did not indicate major violations of linear-regression assumptions, the linear model was retained.

Mean gestation duration for singleton pregnancies (*n* = 36) was 147.42 ± 0.18 days, while twin pregnancies (*n* = 15) averaged 147.93 ± 0.34 days. Male offspring (*n* = 18) were associated with slightly longer gestation (148.17 ± 0.31 days) compared to females (147.24 ± 0.17 days).

Gestation duration and absolute prediction error by litter size and offspring sex are summarized in Table 3. Absolute prediction error was 3.42 ± 0.35 days in singleton pregnancies and 2.33 ± 0.54 days in twin pregnancies. By offspring sex, absolute prediction error was 3.28 ± 0.59 days for male offspring and 3.00 ± 0.34 days for female offspring.

**Table 3:** Gestation duration and absolute lambing-date prediction error according to litter size and offspring sex.

Factor	Category	*n*	Gestation duration (days)	Absolute prediction error (days)
Litter size	Singleton	36	147.42 ± 0.18	3.42 ± 0.35
Litter size	Twin	15	147.93 ± 0.34	2.33 ± 0.54
Offspring sex	Male	18	148.17 ± 0.31	3.28 ± 0.59
Offspring sex	Female	33	147.24 ± 0.17	3.00 ± 0.34

Values are presented as mean ± SE. Absolute prediction error represents the absolute difference between estimated and observed lambing dates.

### 3.7 Natural-mating comparison of lambing-date prediction

The retrospective natural-mating comparison included 49 ewes with complete mating and subsequent lambing records. Mean gestation duration was 147.24 ± 0.28 days. When the predefined fixed 148-day interval was applied, the mean signed prediction error was +0.76 ± 0.28 days, the mean absolute prediction error was 1.37 ± 0.22 days, and the RMSE was 2.06 days. Four of 49 ewes (8.2%) lambed on the exact predicted date, 38 of 49 (77.6%) lambed within ±1 day, and 47 of 49 (95.9%) lambed within ±2 days of the predicted date (Table 4). Overall, prediction errors were concentrated close to the predefined lambing date. All animals meeting the prespecified eligibility criteria were retained in the analysis irrespective of prediction error.

**Table 4:** Prediction performance of the fixed 148-day interval in the natural-mating comparison.

Parameter	Result
Evaluable ewes	49
Gestation duration (days)	147.24 ± 0.28
Signed prediction error (days)	+0.76 ± 0.28
Absolute prediction error (days)	1.37 ± 0.22
RMSE (days)	2.06
Exact predicted date	4/49 (8.2%)
Within ±1 day	38/49 (77.6%)
Within ±2 days	47/49 (95.9%)
Gestation-duration range (days)	146–159

Values are presented as mean ± SE or *n* (%), as appropriate. Signed prediction error was calculated as estimated minus observed lambing date. RMSE, root mean square error. All 49 animals with complete mating and lambing records were retained irrespective of prediction outcome. This retrospective comparison was not considered an independent validation population.

Prediction accuracy was also recalculated using alternative fixed gestation intervals from 146 to 152 days to examine the sensitivity of prediction performance to the assumed gestation length (Table 5). In this retrospective sensitivity analysis, the highest ±1-day accuracy was obtained using a 147-day interval (95.9%), whereas the predefined 148-day interval resulted in 77.6% of lambings within ±1 day and 95.9% within ±2 days. These alternative intervals were evaluated descriptively and were not used to redefine the prespecified 148-day reference.

**Table 5:** Sensitivity of lambing-date prediction accuracy to the fixed gestation interval used in the retrospective natural-mating comparison.

Gestation length used for prediction (days)	Within ±1 day	Within ±2 days	Within ±3 days
146	43/49 (87.8%)	47/49 (95.9%)	47/49 (95.9%)
147	47/49 (95.9%)	47/49 (95.9%)	47/49 (95.9%)
148	38/49 (77.6%)	47/49 (95.9%)	47/49 (95.9%)
149	4/49 (8.2%)	38/49 (77.6%)	48/49 (98.0%)
150	0/49 (0.0%)	5/49 (10.2%)	39/49 (79.6%)
151	1/49 (2.0%)	1/49 (2.0%)	5/49 (10.2%)
152	1/49 (2.0%)	1/49 (2.0%)	1/49 (2.0%)

Prediction dates were recalculated using fixed mating-to-lambing intervals of 146–152 days in the same 49 ewes. The 148-day interval was the predefined reference used in the primary analysis; the alternative intervals are shown only to illustrate how prediction accuracy changes when the assumed gestation length is varied.

## 4 Discussion

This study evaluated whether integrated precision monitoring of maternal, male, and reproductive parameters under commercial flock conditions could support lambing-date estimation and provide biologically meaningful insight into flock-level reproductive dynamics. The integrated system linked verified mating activity, maternal physiological indicators, and ram performance data at the individual-animal level, while the regression analyses identified exploratory associations with prediction error and gestation duration. Accordingly, the main contribution of the study is the integrated reproductive-monitoring framework and the within-flock evaluation of a fixed 148-day prediction rule, rather than a stand-alone validated prediction model.

### 4.1 Maternal body weight and BCS across reproductive stages

No significant differences in body weight or BCS were observed during early gestation or among litter-size categories. The limited variation in BCS may reflect adequate nutritional management and the relatively narrow BCS range of the studied ewes. Moderate BCS at mating has previously been associated with improved reproductive performance in ewes (31), although such an association was not evident in the present early-gestation data. Appropriate nutritional management during late gestation is important for maintaining ewe condition and supporting lamb viability (3, 32). The post-lambing decline in body weight and BCS likely reflects increased energy mobilization during lactation, emphasizing the importance of adequate nutritional support during this period (32, 33). The similar BCS values observed across litter-size categories suggest that BCS alone may not distinguish pregnancy rank under adequately fed conditions, consistent with Pesántez-Pacheco et al. (33).

### 4.2 Synchronization and estrus behavior

The synchronization component illustrates why post-synchronization reproductive intervals must be interpreted in the context of mating exposure. The 16.33-day interval in Group 1 represented return to estrus in nonpregnant ewes after controlled mating, whereas the 19.95-day interval in Group 2 represented the interval from an unmated induced estrus to the subsequent spontaneous estrus. Therefore, the difference should not be interpreted as a direct effect of mating management on cycle length. The numerically longer estrus duration observed during the subsequent spontaneous estrus in Group 2 than during the induced estrus in Group 1 may reflect tighter temporal synchronization of follicular development after FGA–eCG treatment; however, ovulation was not monitored and the difference was not statistically significant, so this interpretation remains tentative. Observed estrus behaviors, including tail wagging, courtship, and standing heat, were consistent with previously described behavioral signs of estrus in small ruminants (27), but they were not associated with pregnancy outcome in this dataset.

### 4.3 Service capacity and reproductive performance

Service capacity was positively correlated with the 45-day pregnancy rate and negatively correlated with sperm concentration. However, because only five rams were evaluated, these associations should be considered exploratory and should not be interpreted as evidence of causality. The absence of significant associations with most other reproductive variables indicates that mating activity and semen characteristics should be interpreted as complementary components of ram reproductive performance. Comprehensive assessment should therefore include physical examination, testicular measurements, semen evaluation, and sexual activity (13, 14).

The inverse association between service capacity and sperm concentration may reflect the effect of more frequent ejaculation in sexually active rams, which can reduce sperm concentration per ejaculate while increasing the number of mating opportunities. Conversely, the positive association with pregnancy rate suggests that sexual activity and mating frequency may contribute to reproductive performance independently of sperm concentration alone. Nevertheless, these mechanisms cannot be confirmed because only five rams were evaluated.

Service capacity is commonly used as a field indicator of ram mating performance and has been associated with flock fertility (34, 35). Seasonal variation in scrotal circumference, semen quality, and sexual activity should also be considered when interpreting ram reproductive assessments (36).

### 4.4 Lambing-date prediction

Accurate lambing-date prediction is important for targeted periparturient monitoring. In the primary analytical dataset, singleton pregnancies were associated with greater absolute prediction error, maternal body weight was inversely associated with absolute prediction error, and male offspring were associated with slightly longer gestation. Because litter size was not significantly associated with gestation duration, the singleton effect is better interpreted as a difference in how closely the fixed 148-day rule fitted this dataset than as evidence of a universal litter-size effect on gestation length. The associations with maternal body weight and offspring sex may reflect differences in maternal energy status, fetal growth, or endocrine maturation, but fetal growth, birth weight, placental function, and endocrine variables were not measured; these mechanisms therefore remain speculative. Previous sensor-based studies have shown that ewe-ram interaction data can support lambing-date prediction (8, 9). Cunningham et al. (9) reported the highest prediction accuracy using intervals of 149–150 days in naturally cycling Merino ewes, with the day of peak ewe–ram interactions used as the temporal reference. In contrast, the present study used the first confirmed mating after FGA-eCG synchronization. Differences in reproductive management and temporal reference may therefore explain why a 148-day rule was a practical within-flock reference here. The regression models nevertheless explained only 20.5 and 15.1% of the variability in absolute prediction error and gestation duration, respectively, and were based on 47 complete cases; their coefficients should therefore be regarded as exploratory rather than as a validated predictive model.

The retrospective natural-mating comparison provided additional within-flock evidence regarding the practical performance of the fixed 148-day interval. Mean gestation duration was 147.24 ± 0.28 days and mean absolute prediction error was 1.37 ± 0.22 days, with 77.6 and 95.9% of lambings occurring within ±1 and ±2 days of the estimated date, respectively. Because the same 148-day interval was applied without modification, these findings provide within-flock support for using this interval to target periparturient monitoring under routine natural-mating conditions. The sensitivity analysis further showed that prediction performance depended on the assumed gestation interval, with 147 days providing the highest ±1-day accuracy in this retrospective dataset. This finding does not invalidate the predefined 148-day reference but indicates that the optimal fixed interval may vary according to flock, reproductive management, season, and the temporal definition of mating. Accordingly, the 148-day interval should be considered a practical reference rather than a universally optimal gestation length. However, this analysis should not be interpreted as independent validation, and the lower prediction error observed in the natural-mating comparison should not be interpreted as evidence that natural mating is superior, because the analytical groups differed in reproductive management, timing of mating, and available records and were not prospectively randomized for this comparison.

### 4.5 Practical implications and limitations

For flock managers, the most immediate value of the integrated system is the ability to link individual identification, confirmed mating records, body-weight data, reproductive examinations, and lambing outcomes within one workflow. This can help define a narrower period for intensified periparturient monitoring at the individual-ewe level. The supplementary handling data also suggest lower time and labor requirements when the smart handling unit was used; however, these operational comparisons were descriptive and were not designed as a formal efficiency trial. The system should therefore be viewed as a decision-support and data-integration tool rather than a stand-alone predictor of parturition.

This study has several limitations that should be considered when interpreting the findings. The work was conducted in a single commercial Merino flock, and therefore the predictive model should be externally validated in different breeds, production systems, and environmental conditions. The number of rams was limited, which may have influenced the strength of correlations involving service capacity and semen traits. Therefore, correlations involving service capacity and semen characteristics should be considered exploratory. The regression models explained only a limited proportion of the observed variability. The *R*^2^ values of 0.205 for absolute lambing-date prediction error and 0.151 for gestation duration indicate that approximately 79.5 and 84.9% of the variability in these outcomes, respectively, remained unexplained. Furthermore, the regression analyses were based on 47 complete cases, below the estimated sample sizes of approximately 52 and 73 ewes required to achieve 80% power based on the observed effect sizes. Accordingly, these regression findings should be considered exploratory. The retrospective natural-mating comparison provides additional within-flock support for the practical use of the fixed 148-day prediction interval; however, it does not constitute independent or external validation. Moreover, mating dates in the natural-mating comparison ranged from June to October, whereas the synchronization component was conducted during October–November. Therefore, seasonal and reproductive-management differences may have contributed to between-cohort variation and preclude direct causal comparison. Unmeasured variation in the actual timing of ovulation and conception, fetal growth, placental function, birth weight, maternal physiology, and environmental conditions may account for additional unexplained variability. External validation in independent flocks, breeds, reproductive seasons, and production systems remains necessary.

No prospectively untreated control group was included in the synchronization component; consequently, the synchronization findings cannot be interpreted as a comparison between hormonally treated and untreated cyclic ewes.

Sheep are seasonal breeders, and reproductive activity may vary according to breed and degree of seasonal response (37); maternal live weight at mating may influence reproductive performance (38), while fetal growth and birth weight are additional biological factors relevant to gestational outcomes (39). The synchronization component was conducted during the natural breeding season (October–November 2022), whereas mating dates in the natural-mating comparison spanned June–October 2022. Although all animals originated from the same commercial flock, this difference in reproductive timing should be considered when interpreting the comparison. For this reason, the proposed approach should be regarded as a decision-support tool rather than a stand-alone predictor of parturition.

## 5 Conclusion

Integrated smart flock monitoring provided a practical framework for linking maternal condition, mating activity, ram performance, pregnancy diagnosis, and lambing outcome at the individual-animal level. In the primary analytical dataset, the mean absolute lambing-date prediction error was 3.23 ± 2.47 days, while the multiple regression models identified associations involving maternal body weight, litter size, and offspring sex but showed limited explanatory power. In the retrospective natural-mating comparison of 49 ewes, application of the same fixed 148-day mating-to-lambing interval resulted in a mean absolute prediction error of 1.37 ± 0.22 days, with 77.6% of lambings occurring within ±1 day and 95.9% within ±2 days of the estimated date. These findings provide within-flock support for the use of a fixed mating-to-lambing interval for targeted periparturient monitoring, while the sensitivity analysis indicates that prediction accuracy varies according to the interval selected and that 148 days should be regarded as a predefined practical reference rather than a universally optimal value. However, the regression findings remain exploratory, correlations involving ram service capacity were based on a limited number of rams, and the natural-mating comparison was retrospective and originated from the same flock. External validation across independent flocks, breeds, seasons, and production systems is therefore required before wider implementation.

## Data Availability

The original contributions presented in the study are included in the article/Supplementary material, further inquiries can be directed to the corresponding author.
