# Supplementary material for: Integrated smart flock monitoring of reproductive parameters for lambing date prediction in merino sheep
Source: Front Vet Sci. 2026 Sep 10;13:1937513. doi: 10.3389/fvets.2026.1937513 (PMC13603478; doi:10.3389/fvets.2026.1937513)
Supplement: Supplementary file 1 [file Data_Sheet_1.docx]

Supplementary Material

**Supplementary Figure S1.** Schematic representation of the smart handling and monitoring unit.

The schematic illustrates the Alligator-based single-animal handling unit used for controlled animal passage, RFID-based identification, body-weight recording, sorting, and digital data registration. The animal-control gates and doors can be operated by one person. During passage, the RFID reader detects the individual ear-tag number, and the Bluetooth data converter transfers body-weight data from the electronic weighing platform to the mobile application within approximately 3 s. Animal identification and body weight are registered automatically. Additional individual information, including body condition score, pregnancy status, fetal number, reproductive examination findings, and other management records, can be entered manually into the same application. Numbers indicate: 1, guillotine entry gate; 2, single-animal waiting area; 3, RFID ear tag; 4, Bluetooth data converter; 5, operator position; 6, RFID reader; 7, mobile application; 8, electronic weighing platform; 9, selection door; and 10, exit gate. The schematic is simplified and is not drawn to engineering scale.

**Supplementary Figure S2.** Descriptive comparison of flock-handling efficiency with and without the smart handling unit.

The figure presents the mean handling time per session, number of workers involved, and number of sheep handled per session. Compared with conventional handling, sessions conducted using the smart handling unit were associated with a lower mean handling time (2.24 ± 1.23 vs. 4.89 ± 2.60 h), fewer workers (3.43 ± 0.84 vs. 5.15 ± 1.68), and a higher number of sheep handled per session (118.46 ± 70.27 vs. 77.33 ± 60.72). These results are descriptive and were not subjected to inferential statistical comparison. Values are presented as mean ± SD. “Without the smart handling unit” refers to conventional flock-handling sessions, whereas “with the smart handling unit” refers to sessions conducted using the Alligator-based system. Although the gates and animal-control components could be operated by one person, the reported workforce values include all personnel participating in the complete management session, including animal examination, data recording, and supporting activities.

**Supplementary Figure S3.** Animal allocation, exclusions, and analytical subsets.

Flowchart showing allocation of the 210 routinely managed Merino ewes to the synchronization and routine-management components, post-exposure reclassification within the synchronized cohort, exclusions from the subsequent-estrus evaluation, and the analytical subsets used for lambing-date prediction and multiple regression. The commercial flock included 11 mature breeding rams, five of which underwent detailed breeding-soundness, semen-quality, and service-capacity evaluations.

**Supplementary Figure S4.** Residual diagnostic plots for the multiple linear regression models.

Residual diagnostic assessment of the multiple linear regression models. (A) Absolute lambing-date prediction error model. (B) Gestation duration model. Standardized residuals showed no clear pattern against predicted values, and normal Q–Q assessment indicated approximate normality. Durbin–Watson statistics were close to 2, variance inflation factors were below the accepted threshold, and no observations showed excessive leverage or Cook’s distance.
